# Management of spinal infection: a review of the literature

**DOI:** 10.1007/s00701-018-3467-2

**Published:** 2018-01-22

**Authors:** Sara Lener, Sebastian Hartmann, Giuseppe M. V. Barbagallo, Francesco Certo, Claudius Thomé, Anja Tschugg

**Affiliations:** 1 Department of Neurosurgery, Medical University lnnsbruck, Anichstrasse 35, A-6020 Innsbruck, Austria; 2 Department of Neurosurgery, Policlinico “G. Rodolico” University Hospital, Catania, Italy

**Keywords:** Spinal infection, Spondylodiscitis, Vertebral osteomyelitis, Spinal epidural abscess, Intramedullary abscess, Subdural empyema

## Abstract

Spinal infection (SI) is defined as an infectious disease affecting the vertebral body, the intervertebral disc, and/or adjacent paraspinal tissue and represents 2–7% of all musculoskeletal infections. There are numerous factors, which may facilitate the development of SI including not only advanced patient age and comorbidities but also spinal surgery. Due to the low specificity of signs, the delay in diagnosis of SI remains an important issue and poor outcome is frequently seen. Diagnosis should always be supported by clinical, laboratory, and imaging findings, magnetic resonance imaging (MRI) remaining the most reliable method. Management of SI depends on the location of the infection (i.e., intraspinal, intervertebral, paraspinal), on the disease progression, and of course on the patient’s general condition, considering age and comorbidities. Conservative treatment mostly is reasonable in early stages with no or minor neurologic deficits and in case of severe comorbidities, which limit surgical options. Nevertheless, solely medical treatment often fails. Therefore, in case of doubt, surgical treatment should be considered. The final result in conservative as well as in surgical treatment always is bony fusion. Furthermore, both options require a concomitant antimicrobial therapy, initially applied intravenously and administered orally thereafter. The optimal duration of antibiotic therapy remains controversial, but should never undercut 6 weeks. Due to a heterogeneous and often comorbid patient population and the wide variety of treatment options, no generally applicable guidelines for SI exist and management remains a challenge. Thus, future prospective randomized trials are necessary to substantiate treatment strategies.

## Introduction

Spinal infection (SI) is defined as an infectious disease affecting the vertebral body, the intervertebral disc, and/or adjacent paraspinal tissue [61]. It comprises infection due to a contiguous source (trauma, surgery) as well as due to hematogenous dissemination [13]. SI represents 2–7% of all musculoskeletal infections. There is a bimodal allocation in patients suffering from SI, with one peak below 20 years and the other between 50 and 70 years of age [48, 77]. Gender-specific investigations report a male/female ratio ranging between 2:1 and 5:1 [35, 59]. The incidence varies between 1:20,000 and 1:100,000, and mortality rates range between 2 and 20% in developed countries [1, 7, 44, 70]. However, the incidence of SI has been increasing in the last decades [78]. This may be related to improving imaging techniques and clinical diagnostics, to the progressive number of older patients suffering from chronic diseases, to an increased amount of intravenous drug abusers, and to the growing number of spinal surgery and instrumentation [17]. Patients suffering from SI commonly present with unspecific symptoms, back pain being the most frequently reported (85%), followed by fever (48%) and paresis (32%) [23, 25, 59]. Due to the unspecific presentation, the early detection of SI is still challenging and sufficient treatment is complex.

## Pathophysiology and classification

**Fig. 1:**
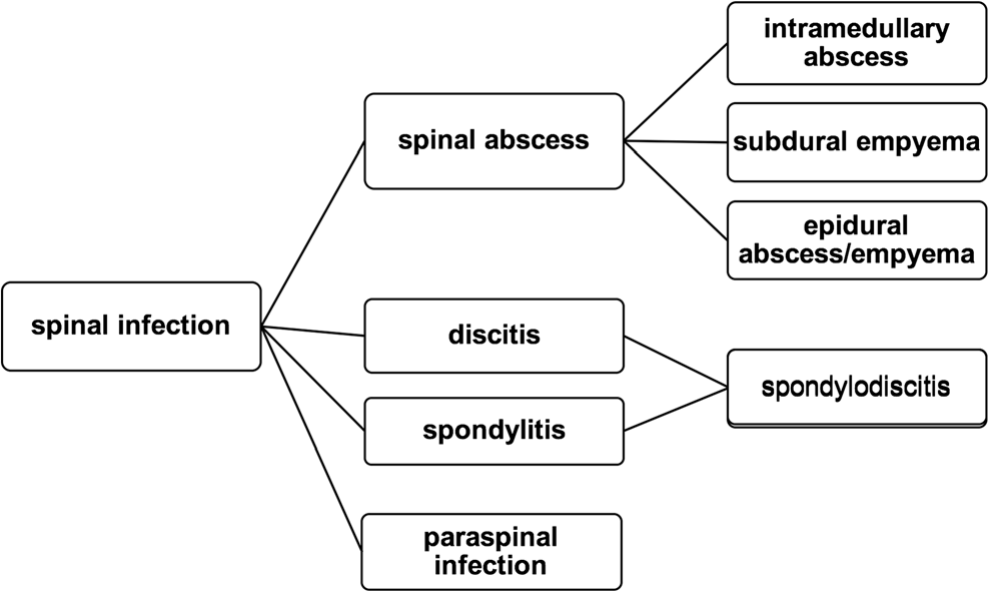
Classification of spinal infection based on the localization of the infection and the affected tissue

**Fig. 2:**
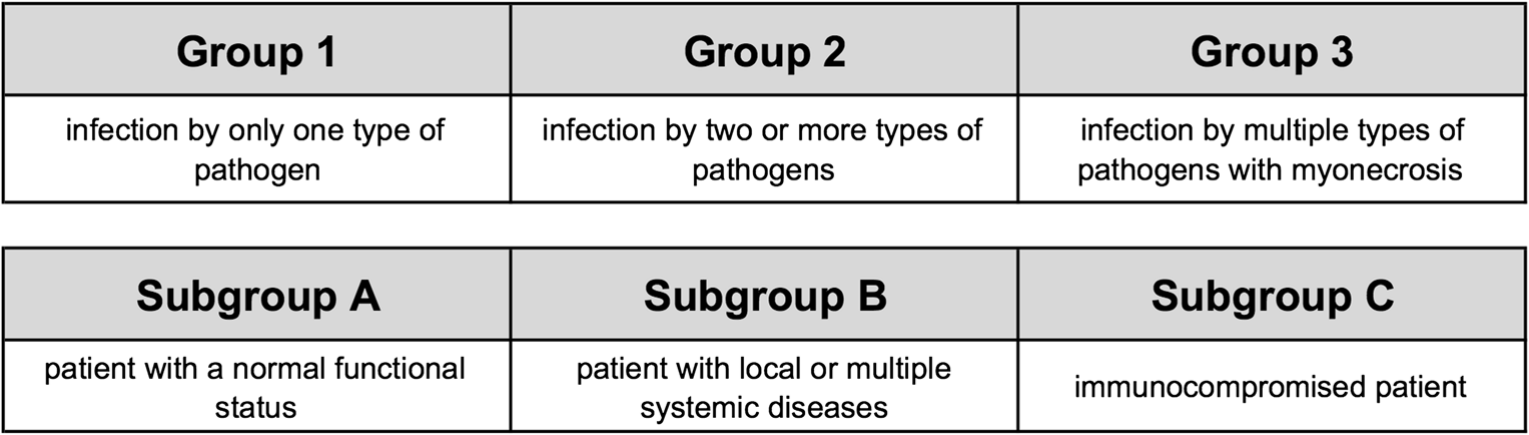
Classification of the types of infection by Thalgott (1991). The aim of the grading is to gauge the severity of the (postoperative) spinal infection and its risk factors [79]

Basically, SI can be caused from hematogenous spread from a distant site, by dissemination from contiguous tissues or by direct external inoculation. In adults, discitis mostly originates from one of the neighboring endplates, which are necrotized by a septic embolus, while the disc is infected secondarily [65]. Differently, the origin in infantile discitis is controversial, as the perfusion of the disc is obtained by anastomoses of the intraosseous arteries and small vessels penetrating the disc. Therefore, a septic embolus does not cause prior bone infarction, and the infection is primarily located in the infantile disc [65]. Pyogenic spondlylodiscitis through hematogenous spread mostly affects the lumbar spine (58%) followed by the thoracic (30%) and cervical spine (11%) [32, 65]. This differs from tuberculous lesions, which mainly affect the thoracic spine and often more than two levels, which can be a distinctive feature to pyogenic infection [65]. Nevertheless, a spinal infection can lead to uncontrolled spread beyond bony structures and affect the surrounding tissues. Spread from contiguous tissue is rare and mainly occurs in adjacent infection, including retropharyngeal abscess, esophageal ruptures, and infected implants [9]. The way of direct external inoculation is mostly iatrogenic and follows infiltrations or surgical procedures [74]. The classification of SI is primarily done by the location of the infection, roughly discerning between intraspinal infection, bone infection, disc (and bone) infection, and paraspinal infection (i.e., psoatic abscess) and is outlined in more detail in Fig. 1. Another, less common way to classify (postoperative) SI is based on the number of isolated microorganisms (see Fig. 2). The aim of this classification is to simplify the choice of treatment by weighing the severity of the SI and its risk factors.

## Risk factors

There are numerous factors which may facilitate the development of SI, only some of which can be influenced [47]. Non-influenceable risk factors include but are not limited to advanced patient age, American Society of Anaesthesiologists (ASA) score, and comorbidities like obesity, diabetes mellitus, substance abuse, chronic infection (especially HIV), long-term systemic steroid use, a poor nutritional status, and immunologic incompetence [5, 17, 18]. The most important influenceable factor promoting SI is spinal surgery. Long duration of surgery, high blood loss, type of instrumentation (lumbar and posterior more frequently than cervical and anterior [16]), and the quantity of operations (revisions, multiple interventions) are significant risk factors, leading to a SI rate of 1–4% after spinal surgery [46]. Particular attention should be paid to surgically treated obese patients, as a subcutaneous fat thickness > 50 mm may lead to a significantly higher risk of postoperative infection and colonization by microorganisms [52].

## Microbiology

Spinal infections are caused by three major agents: bacteria—causing pyogenic infection, fungi—causing granulomatosis infection, and parasites, which are rare. The etiology of SI varies depending on the location of the infection, as different pathogens affect different localizations. *Staphylococcus aureus* is the most common agent when it comes to spinal epidural abscesses (30–80%). Further frequent pathogens are coagulase-negative Staphylococci and several species of Streptococci, which are also involved in spondylitis/spondylodiscitis and paraspinal abscesses. Likewise, *Escherichia coli* frequently causes spondylitis/spondylodiscitis and paraspinal infections. Fungal causes of SI are rarely found and particularly include Aspergillus spp., Candida spp., and *Cryptococcus neoformans*. Although analysis has improved and identification of pathogens is highly pursued, in one third of cases, no organism can be identified [33, 68].

## Diagnosis

Patients suffering from SI present with an insidious onset and unspecific symptoms including neck/back pain, fever (even though not in an early stage), painful dorsal flexion, and, contingently, neurological deficits [23, 25, 59]. Early diagnosis is hindered by the fact that 30–70% of patients with spondylitis/spondylodiscitis do not show any signs of prior infection [23]. Due to the low specifity of signs, the delay of diagnosis remains an important issue. Even though diagnostic tools and procedures have improved, a delay of 2 to 6 months between first symptoms and diagnosis is reported. As a result, poor outcome is frequently seen [12, 28, 82]. Diagnosis should be supported by clinical, laboratory, and imaging findings [12, 28].

## Laboratory parameters

Several clinical routine markers are suitable for diagnosis and evaluation of treatment response. The C-reactive protein (CRP) is considered to be the most specific marker for treatment response, as it returns to normal levels rapidly after successful treatment. Furthermore, CRP is elevated in more than 90% of acute spondylodiscitis cases and is a sensitive marker for bacterial infection. The erythrocyte sedimentation rate (ESR) also is a sensitive marker for infection but has low specificity. It can be used as a marker for therapeutic response only to some degree, as ESR is still increased in 50% of patients with good clinical outcome [14]. The white blood cell (WBC) count, however, is less useful than ESR and CRP, as the presence of a normal WBC count does not exclude the diagnosis of spinal infection [19]. Procalcitonin (PCT), a promising marker to distinguish between bacterial and non-bacterial infection, shows lower sensitivity than CRP in patients with SI. With multiple infected sites, its sensitivity increases. Thus, patients with elevated PCT levels should be considered as suffering from combined infection, and sufficient antibiotic treatment is required [41]. As soon as SI is suspected in a patient, blood and urine cultures should be collected before starting an empiric antibiotic therapy [54]. Up to 59% of positive blood cultures identify the etiological microorganism in patients with monomicrobial pyogenic spondylodiscitis [77]. Based on the fact that biopsy may be superior to blood culture, computed tomography (CT)-guided biopsy of infected tissue should be considered [29].

## Imaging

Magnetic resonance imaging (MRI) remains the most reliable method to diagnose spondylodiscitis, due to its high sensitivity (96%), high specificity (94%), and capability to provide detailed data on paraspinal tissues and the epidural space [51, 53, 57]. Typical findings in patients with spondylodiscitis are hypointense discs and vertebral bodies in T1-weighted images and hyperintense signals of the same structures in T2-weighted images.

Nevertheless, plain and flexion/extension X-rays should be performed in every baseline evaluation. However, the specificity of native X-rays in the diagnosis of spondylodiscitis is low (59%) and can at most detect irregularities of vertebral endplates and/or low intervertebral disc height in advanced cases [42]. A possible instability during follow-up can be detected reliably by flexion/extension films. Bony changes, i.e., early changes of endplates, and bone necrosis can be detected most precisely by CT. However, it may take 3 to 6 weeks after the onset of symptoms for bony destruction to become evident, which entails a delay in diagnosis, and makes the CT a less expressive diagnostic instrument for SI [42, 85]. Nevertheless, CT is commonly used in CT-guided biopsy and prior to instrumentation procedures [20]. Although MRI is the gold standard in diagnosis of SI, there is no pathognomonic finding on MRI that dependably discriminates between SI and possible neoplasm. In case of suspicion of SI, complementary diagnostic methods can be applied [23]. Sequential bone/gallium imaging and 67Ga-SPECT constitute radionuclide options for SI. Since these two methods are characterized by low specificity, interest increased in fluoro-2-desoxy-D-glucose [18F] PET, which may be a promising technique for distinction, as degenerative changes and fractures normally do not demonstrate FDG uptake [30, 31]. At present, however, radionuclide diagnostics should be reserved for cases of uncertain diagnosis or for special follow-up [23].

Since positive blood cultures and imaging findings associated with clinical symptoms do not automatically confirm SI, the definite diagnosis can only be attained by microscopic or bacteriological examination of the affected tissue. Both blood cultures and imaging can be inconclusive or false negative, so that a definite confirmation of SI diagnosis should be aimed for to ensure the quality of management [24, 37, 43, 84].

## Management

### Spinal abscess

#### Intramedullary abscess

Intramedullary spinal cord abscesses (ISCA) are very rare with about 120 reported cases since the first description in 1830 [49]. Congenital dermal sinus is implicated as the leading cause of ISCA in children, whereas hematogenous spread of urogenital and lung infections or infective endocarditis is the most common cause in adults. Usually some underlying spinal (cord) pathology provides the basis for ISCA [80]. The overall mortality of ISCA has improved significantly from 90% in 1944 [8] to 4% in 2009 [49], mostly caused by the availability of sufficient diagnostic facilities, the use of antibiotic agents, and improved surgical techniques. Due to the small number of cases, there is no general algorithm for ISCA and treatment has to be chosen diligently according to the patient’s clinical status. Generally, early drainage and the rapid administration of intravenous antibiotics result in a good prognosis [40]. Surgical drainage within 5 days after onset of symptoms may lead to a significantly better neurological outcome than conservative treatment or delayed drainage [40, 75].

#### Subdural empyema

Subdural empyema (SDE), known as an infection located between the dura and the arachnoid, is a very rare condition and mostly results from hematogenous infection or spread of infection from osteomyelitis [36]. SDE may develop in the spinal canal where it is possible to cause rapid compression of the spinal cord. The diagnostic procedure of choice is MRI with gadolinium, followed by CT with myelography [50, 63]. Early surgical drainage followed by appropriate antibiotic therapy is the most promising treatment [10]. Depending on the extension of the lesion, (hemi-)laminectomy over one or more levels may be necessary. In cases of wider spread, flavectomy or laminotomy at several different levels may be required in order to evacuate the infectious material [2, 50]. Generally, SDE represents an extreme medical and neurosurgical emergency and treatment requires prompt surgical drainage and antibiotic therapy, as morbidity and mortality in SDE relate directly to treatment delay. As soon as the central nervous system is directly involved, outcome is poor [69]. Thus, the condition should be treated with great urgency [36].

#### Epidural abscess/empyema

Spinal epidural abscess (SEA) also is a life-threatening infection. Mortality rates are estimated at 5–16% worldwide, and less than 50% of surviving patients show full recovery. Although SEA is a relatively rare diagnosis (2.5–3.0/100,000), the incidence has nearly doubled in the last 50 years according to circumstances already mentioned above and an increasing incidence of SI in general [6, 71]. Demographically, male patients are affected more frequently than female patients (ratio 2:1) for unknown reasons. Furthermore, a peak is reported in the sixth decade of life [66]. Spontaneous SEA is mostly a secondary complication of a primary SI like spondylodiscitis, spreading hematogenously via septic thrombosis of epidural veins [37]. Alternatively, iatrogenic SEA is mainly associated with surgical spinal procedures and in particular with epidural anesthesia or spinal injections [56]. Abscesses are most commonly located in the lumbar spine (48%), followed by the thoracic (31%) and the cervical spine (24%) [6]. Mostly, SEAs present as a multisegmental (three to four segments) disease as germs are capable of spreading via the epidural space without anatomical resistance. SEA is found in multiple locations in 20% of cases; therefore, imaging of the whole spine is recommended [19]. As soon as an active infection is suspected, treatment should be started immediately. Decompression combined with systemic antibiotics has been established as gold standard in the last decades, especially in patients with progressive infection and late diagnosis [6]. Nevertheless, management should be guided by individual parameters, as recently good outcomes were also demonstrated for conservative treatment. Conservative management can be considered (1) in patients with severe comorbidities which limit surgical options, (2) in extremely lengthy SEA (holospinal), in which surgery may not make sense or may be unachievable, (3) when SEA is detected early and patients suffer from no or only minor neurologic deficits, or (4) when the patient presents with complete paralysis for more than 72 h [66]. However, 6–49% of initially conservatively treated patients still show progression of neurologic deficits, so that treatment has to be reconsidered carefully after meticulous re-evaluation [6, 19]. Special attention should be paid to patients suffering from high-risk factors for failure of medical treatment. Those include diabetes mellitus, MRSA infection, neurological impairment involving the spinal cord, acute or progressive deficit, CRP > 115 mg/L, WBC count > 12.5 × 10^9^ cells/L, ring-like enhancement on MRI, and bacteraemia. Non-operative treatment should only be offered with close monitoring as the initial line of treatment and does not guarantee the prevention of operative therapy [83]. Surgical options for the treatment of SEA include minimally invasive or endoscopic procedures, multisegmental decompression, instrumentation, and ventral debridement of the disc. The choice of the surgical approach depends on the consistency of the space-occupying lesion: solid (granulation tissue) or liquid (abscess). Dorsally accessible abscesses call for a minimally invasive drainage via laminotomy and irrigation, whereas dorsally located granulation tissue should be treated with multisegmental decompression and/or resection. Apart from that, ventrally located granulation tissue, which is usually associated with spondylodiscitis, is most effectively treated by corpectomy, reconstruction, and instrumentation [15]. Overall, patients being surgically treated show a good outcome in more than 60%. Those convincing results cause some authors to recommend surgical therapy of SEA even without associated neurological deficits and especially when lesions are located in the cervical or thoracic spine. As mortality of SEA is still reported to be 5–20%, therapeutic decisions have to be made promptly and thoughtfully and influencing outcome predictors have to be considered. It has to be realized that SEA treated with medical management alone has a high risk for failure, if the patient is older than 50 years and suffers from severe comorbidities like diabetes or MRSA infection and neurologic compromise. In the absence of these risk factors, non-operative management may be considered as the initial line of treatment, but only with very close monitoring [45, 64]. Nevertheless, surgery should stay the treatment of choice in any case of doubt.

#### Spondylodiscitis

**Fig. 3:**
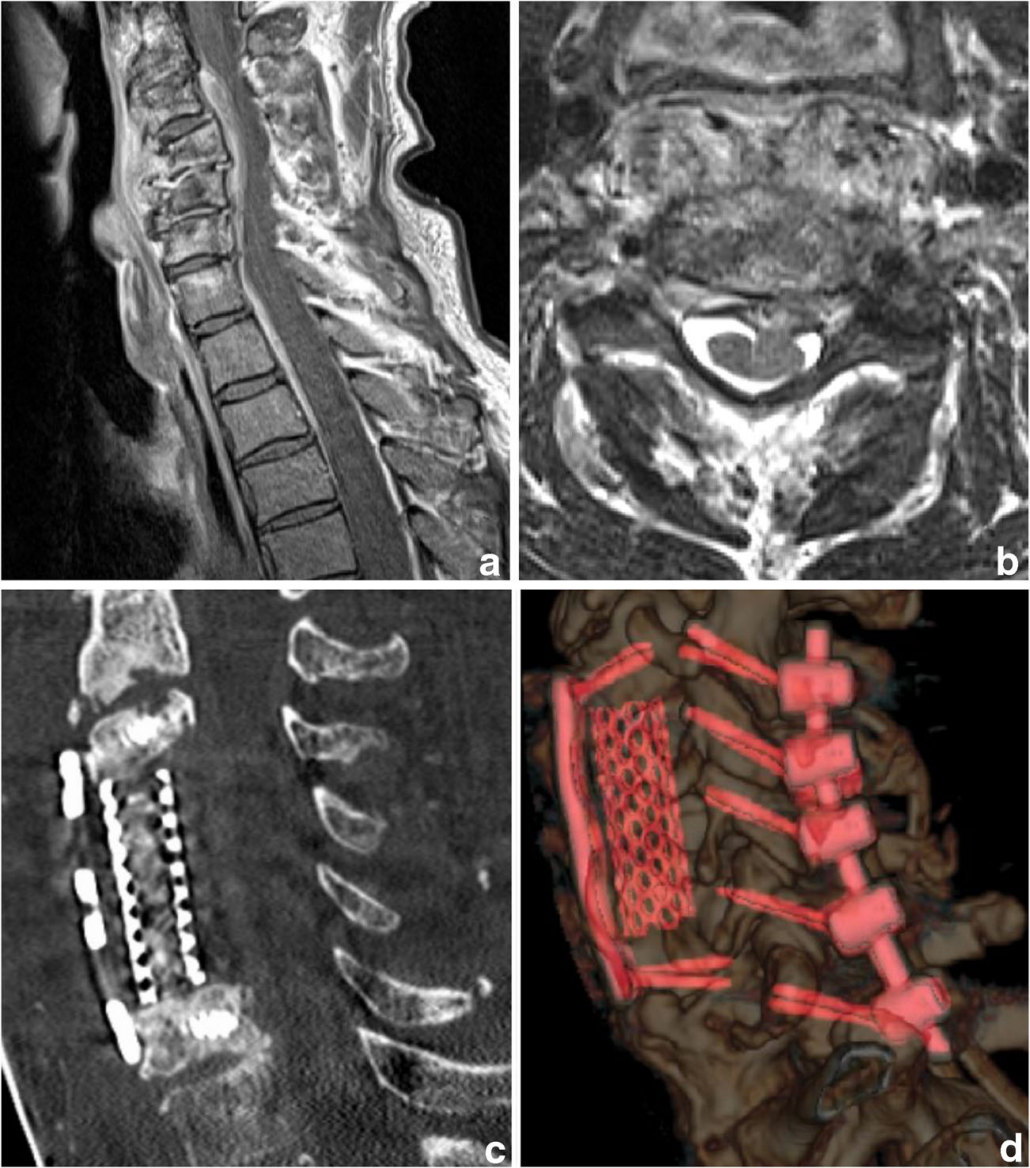
Male patient, 61 years old, suffering from a septic arthritis of the left ankle joint, chronic alcoholism abuse, presenting with a weakness for abduction (3/5) of the left upper extremity for 2 weeks. Laboratory results show elevated inflammatory parameters (CRP: 5.0 mg/dl, leukocytes: 13.0) Blood cultures reveal *Staphylococcus aureus*, and Amoxicillin 2.2 g 3× is started. Imaging depicts a spondylodiscitis C4/5 and C5/6 accompanied by intraspinal and prevertebral abscess, plus kyphotic deformity of the cervical spine. (see image **a** and **b**) A (1) corporectomy with anterior plating C3–6 and vertebral body replacement (image **c**) plus (2) dorsal stabilization C3–7 was performed. (image **d**) Intraoperative biopsy confirms Staph. aureus, Imipenem + Fosfomycin therapy is continued and inflammatory parameters decrease

**Fig. 4:**
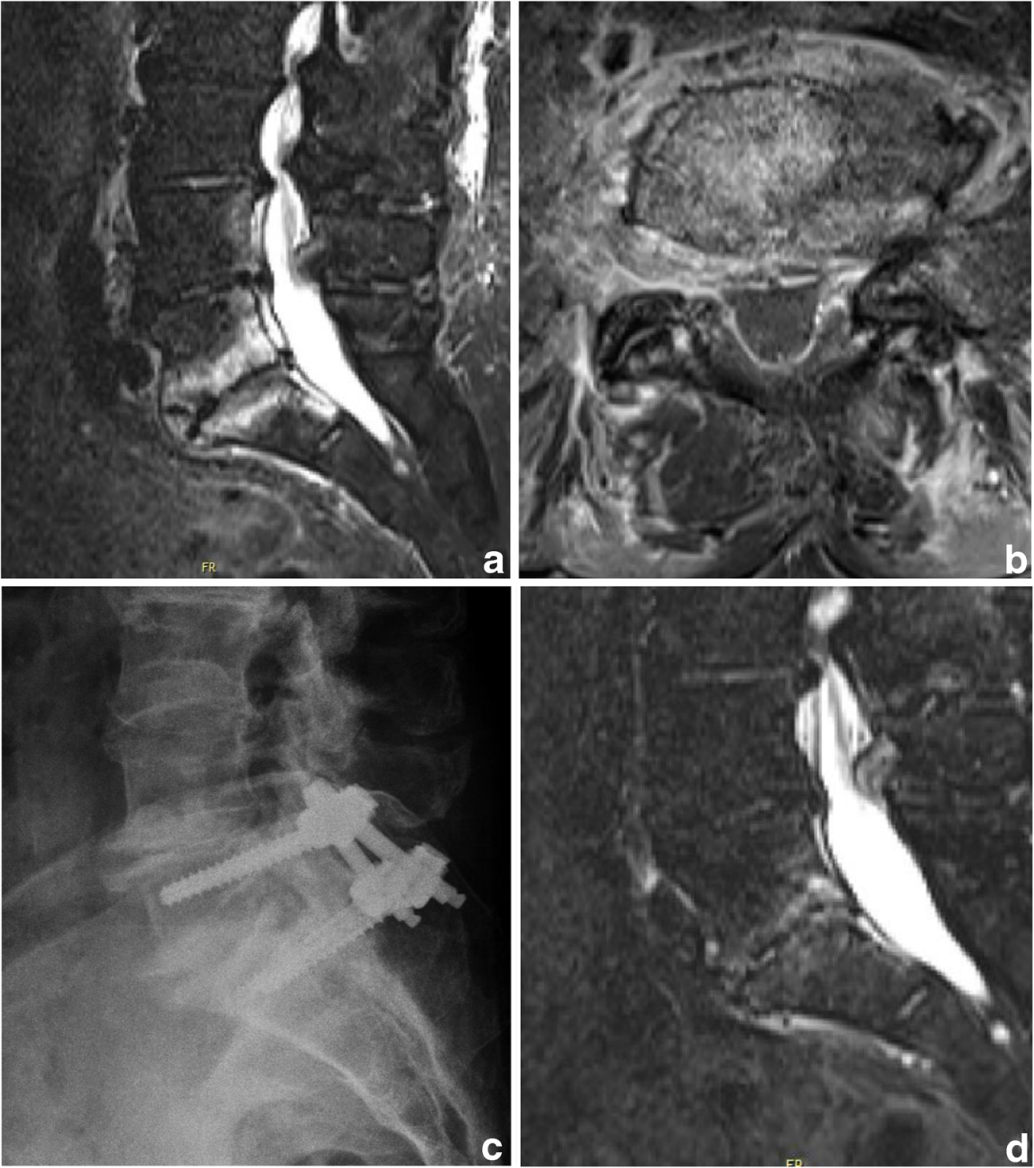
Female patient, 73y, suffering from acute myeloid leukemia (AML), presenting with refractory low back pain following a periradicular infiltration 1 month previously. CRP is elevated (15.8 mg/dl), leukocytes are low due to AML (4.1) and blood culture results are negative. Imaging is highly suspicious for spondylodiscitis L5/S1 with a prevertebral abscess (image **a** and **b**). Percutaneous instrumentation (L5/S1) is performed (image **c**) and empiric antibiotic therapy is started (Dalacin + Ciproxin). CRP decreases over time (3.8 mg/dl 2 weeks after instrumentation) and a complete remission of the symptoms after 2 months (image **d**) is achieved

Spondylodiscitis is defined as an infection of the disc and the adjacent vertebrae, mostly (55–80%) caused by *Staphylococcus aureus* and spread hematogeneously [20]. The infection can emerge primarily or secondarily, due to spinal interventions, whereas primary acquired spondylodiscitis shows a more severe course and significantly higher mortality rate (12.5 vs. 1.8%) than postoperative infections [81]. Incidence ranges from 0.2 to 2.4/100,000 in western countries [15, 32], and male patients are affected twice as often as females [87]. At clinical presentation, the average age is the fifth to sixth decade, and symptoms are frequently unspecific. Unfortunately, this often leads to a delay in diagnosis and may explain why mortality still ranges between 2 and 20% [76, 87]. Hence, spondylodiscitis has to be seen as a life-threatening condition and treated as an emergency. Although some guidelines for treatment are available, therapy for spondylodiscitis is not standardized and based on individual preferences. In general, treatment goals have to predominantly include the control of the infection by treatment of the causative systemic disease and removal of the septic focus. The first-line treatment of choice is a conservative attempt, which is absolutely reasonable in early stage with no, or minor, neurologic deficits and in case of severe comorbidities, which limit surgical options. Initially, Clindamycin + Ciprofloxacin or Cefotaxim + Flucloxacillin are highly recommended to cover a preferably wide spectrum of potential pathogens [27]. Subsequently, therapy has to, of course, be adjusted to results of bacteriologic test results. Appropriate antibiotics should be applied intravenously for 2–4 weeks or until CRP has largely dropped. Thereafter, oral antibiotic treatment is continued for a total of 6 to 12 weeks [27, 76]. Duration and route of administration of antibiotic treatment are still matter of debate, since a relationship between treatment duration and relapse/failure is assumed. Additionally, conservative treatment should include bed rest and/or orthosis for at least 6 weeks, depending on pain upon mobilization [15]. Conservative therapeutic options are successful in many cases, but are not always sufficient. Surgery has to be considered when medical options have failed and symptoms or imaging findings show progress. Some cases may initially require surgical debridement and stabilization. Rapid surgical treatment is mandatory if patients present with advanced stage spondylodiscitis, neurologic deficits, progressive septicaemia, and/or progressive instability or deformity (Table 1). If mobilization fails due to pain in conservatively treated patients, many surgeons nowadays proceed with surgery, as complications of immobilization can be critical in this comorbid population. Classically, an anterior open debridement and, mostly, instrumentation have been the preferred treatment, associated with good outcome and low intraoperative complication rates [32]. Recently, promising techniques improving outcome and lowering complications have been described. Particularly in cervical spondylodiscitis, a single anterior approach has become more and more applicable [67, 73]. However, in cases of extensive involvement requiring multiple corpectomies, a combined anterior and posterior approach is appropriate (Fig. 3). In clinical situations, in which an anterior approach is contraindicated, posterior transforaminal or posterior interbody debridement and fusion can be considered, mostly in cases of lumbar discitis and/or minimal vertebral involvement [26]. Moreover, posterior stabilization and interbody fusion are feasible in elderly high-risk patients with spondylodiscitis and septic complications. Even though it may not be the therapy of first choice in high-risk septic patients, it may be considered in patients when conservative management has failed [38]. Attention should be paid to the method of interbody fusion, as a direct correlation of autologous graft infection to comorbidities and patient’s age has been shown [62]. Minimally invasive disc debridement may lead to immediate pain reduction and good clinical results in patients who suffer from comorbid medical problems and pyogenic spondylodiscitis [39]. Even minimally invasive combined approaches for instrumentation lead to good outcomes in suitable patients, as the technique results in little surgical trauma, intraoperative blood loss, and few postoperative complications. Therefore, the approach is effective and safe for the treatment of single-level lumbar pyogenic spondylodiscitis and could be an alternative to conventional open surgery [55]. Overall benefits of minimally invasive debridement and instrumentation include less blood loss, less spread of infection, less wound infection, and may ultimately lead to more rapid fusion and therefore to faster recovery. Percutaneous instrumentation may only be a valuable option in patients showing no space-occupying abscess and lacking deformity and/or pathologic fracture (Fig. 4) [21]. Percutaneous pedicle screw instrumentation (1) without any debridement [21], (2) with minimally invasive decompression and debridement, or (3) with a second anterolateral debridement/corpectomy is increasingly used [78].

**Table 1:** Established surgical treatment options for spondylodiscitis

1	Minimally invasive/endoscopic debridement
2	Percutaneous instrumentation without debridement
3	Decompression and debridement plus instrumentation
4	Discectomy/corpectomy plus instrumentation
5	Complex anteroposterior reconstruction plus instrumentation

Additional antibiotic treatment (modified to intraoperative culture results) has to be administered for at least 6 to 12 weeks in all cases [27]. The final result in conservative as well as in surgical treatment always is bony fusion, so that conservative management does not preserve segmental motion.

### Paraspinal abscess

Extension of infection into the paraspinal tissues is commonly addressed by treating the underlying spinal infection. If surgical debridement of, e.g., spondylodiscitis is performed, adjacent anterior and lateral abscesses are usually drained as well. Antibiotic treatment reaches the paraspinal tissues generally easier than the non-vascularized disc space. Only in the case of larger abscess formation percutaneous CT- or ultrasound-guided drainage may be indicated [58].

#### Iliopsoas abscess

The most common paraspinal site of infection, the iliopsoas abscess (IPA) can be subdivided in primary and secondary acquired disease. Primarily arisen IPA is a rare condition and mostly occurs due to hematogenous or lymphatic spread of a pathogen from a distant infectious site. On the contrast, the majority of cases seen represent secondary IPA, most commonly caused by inflammatory spinal or skeletal pathology [72]. The recommended first-line therapy is broad spectrum antibiotic therapy, covering *Staph. aureus* as the most frequent pathogen accountable for IPA [60]. Especially small abscesses (< 3 cm) may be treated efficiently with antibiotic therapy alone [86]. In the case of progress or insufficient medical therapy, image-guided percutaneous drainage is a very effective and safe alternative to the open surgical drainage [22]. Nevertheless, in secondarily acquired, multiloculated IPA accompanied by florid spinal infection, open surgical drainage plus treatment of the underlying cause is the treatment of choice [58].

## Discussion

**Fig. 5:**
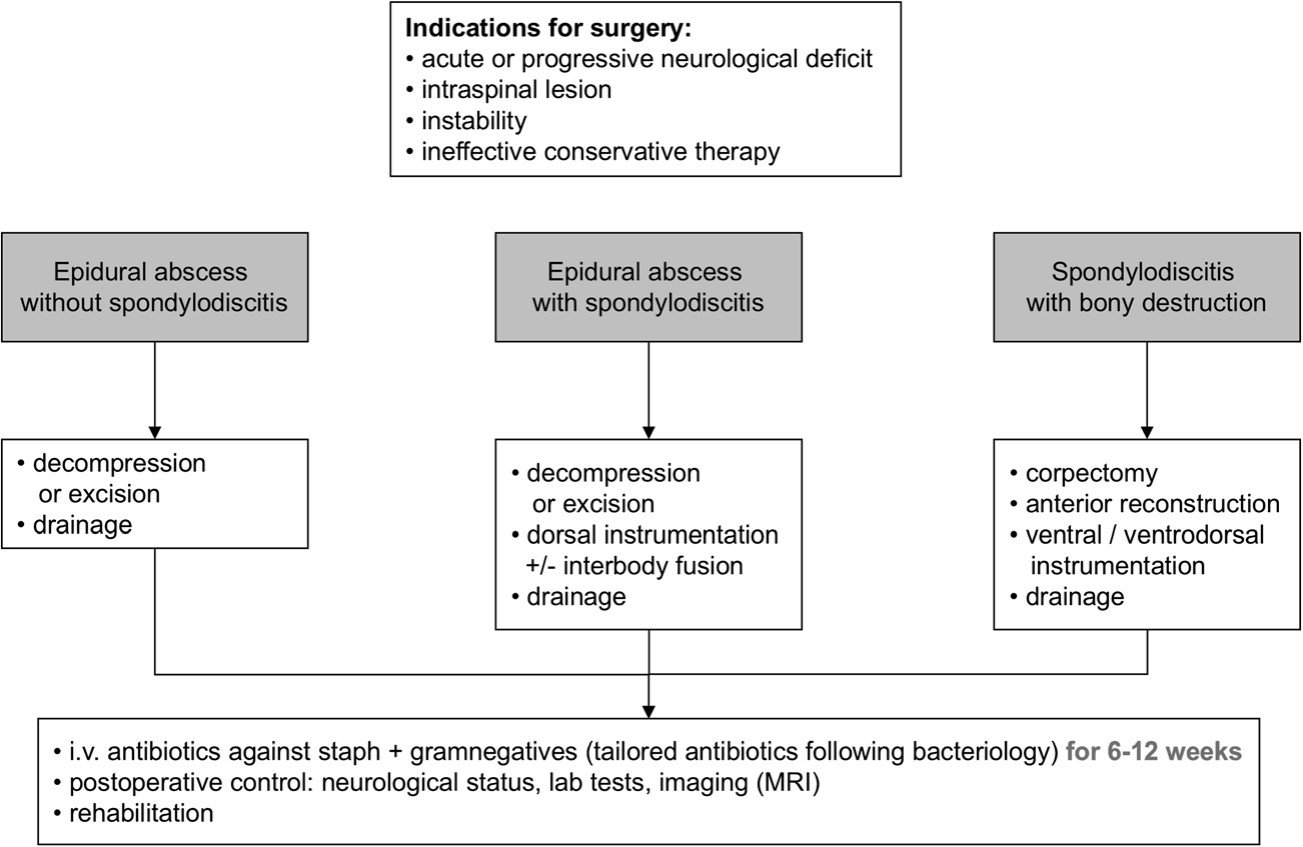
Operative treatment algorithm for SI, modified according to Stuer et al. and Hadjipavlou et al. [37, 78]

Treatment strategies of SI still remain controversial. Conservative cases seem to be followed by mechanical back pain more often than surgical cases [67] and develop more deformity at long term. This comes at a price, as perioperative complication rates are higher in surgically treated cases, although overall mortality is lower in operated patients [78]. Delayed (surgical) treatment entails significantly poorer surgical outcome [4, 19]. Even multimorbid patients with advanced age may show better overall outcomes when treated surgically, despite risking a higher complication rate. The latter can be minimized by choosing the most appropriate, and ideally a minimally invasive, approach. SI patients are more likely “too sick not to do surgery” than inversely. Algorithms like in Fig. 5 have been reported [3, 78]. Among others, the optimal duration for antimicrobial treatment is still unclear, in surgical as in solely conservative treatment. Observational studies reported a significantly higher recurrence rate for treatment duration of less than 8 weeks, compared to antibiotic treatment enduring longer than 12 weeks (10–14 to 3.9%) [34]. Nevertheless, the only randomized controlled trial on the topic ascertained no differences in the outcome after administration of 6 and 12 weeks of tailored antibiotic treatment, so that shorter medical treatment seems to be sufficient in most cases [11]. Consequently, the duration of antibiotic treatment in SI should never remain less than 6 weeks from diagnosis, continued until CRP levels normalize and according to the patient’s overall response [76].

## Conclusion

Due to a heterogeneous and often comorbid patient population and the wide variety of treatment options, no generally applicable guidelines for SI exist and management remains a challenge. In synopsis of the reviewed articles and personal experience, medical and surgical options should always be considered, but the authors favor surgical treatment in case of doubt or progression. With the increasing incidence of spinal infection particularly in elderly and frail patients, our personal opinion supports early surgery, as many patients are admitted in the preseptic phase and often deteriorate in their general condition thereafter. What is more, in the early stage of infection, less invasive procedures may still be possible, while extensive resections may be required at later stages, which are commonly characterized by massive bony destruction. Even in severely affected patients with extensive infection and exceedingly high CRP levels, the authors nowadays tend towards surgery, if medically feasible. Nevertheless, future prospective randomized trials are necessary to substantiate treatment strategies.

## Abbreviations

ASA: American Society of Anaesthesiologists
CRP: C-reactive protein
CT: Computed Tomography
ESR: Erythrocyte sedimentation rate
FDG: Fluorodeoxyglucose
HIV: Human immunodeficiency virus
IPA: Iliopsoas abscess
ISCA: Intramedullary spinal cord abscess
M.: Mycobacterium
MRI: Magnetic resonance imaging
PET: Positron emission tomography
PCT: Procalcitonin
SDE: Subdural empyema
SEA: Spinal epidural abscess
SI: Spinal infection
Staph.: Staphylococcus
SPECT: Single photon emission computed tomography
WBC: White blood cell
